# Clonal interference and changing selective pressures shape the escape of SARS-CoV-2 from hundreds of antibodies

**DOI:** 10.1093/ve/veaf104

**Published:** 2026-02-04

**Authors:** Hugh K Haddox, Omar Abdel Aziz, Jared G Galloway, Javen Kent, Cameron R Cooper, Chris Jennings-Shaffer, Will Dumm, Seth D Temple, Jesse D Bloom, Frederick A Matsen

**Affiliations:** Computational Biology Program, Fred Hutchinson Cancer Center, 1100 Fairview Ave N, Seattle, WA 98109, United States; Computational Biology Program, Fred Hutchinson Cancer Center, 1100 Fairview Ave N, Seattle, WA 98109, United States; Computational Biology Program, Fred Hutchinson Cancer Center, 1100 Fairview Ave N, Seattle, WA 98109, United States; Computational Biology Program, Fred Hutchinson Cancer Center, 1100 Fairview Ave N, Seattle, WA 98109, United States; Morehouse College, 830 Westview Dr SW, Atlanta, GA 30314, United States; Computational Biology Program, Fred Hutchinson Cancer Center, 1100 Fairview Ave N, Seattle, WA 98109, United States; Morehouse College, 830 Westview Dr SW, Atlanta, GA 30314, United States; Computational Biology Program, Fred Hutchinson Cancer Center, 1100 Fairview Ave N, Seattle, WA 98109, United States; Computational Biology Program, Fred Hutchinson Cancer Center, 1100 Fairview Ave N, Seattle, WA 98109, United States; Computational Biology Program, Fred Hutchinson Cancer Center, 1100 Fairview Ave N, Seattle, WA 98109, United States; Department of Statistics, University of Washington, 1410 NE Campus Pkwy, Seattle, WA 98195, United States; Department of Statistics, University of Michigan, 1085 S University Ave, Ann Arbor, MI 48109, United States; Michigan Institute for Data & AI in Society, University of Michigan, 500 Church Street, Ann Arbor, MI 48109, United States; Computational Biology Program, Fred Hutchinson Cancer Center, 1100 Fairview Ave N, Seattle, WA 98109, United States; Basic Sciences Division, Fred Hutchinson Cancer Center, 1100 Fairview Ave N, Seattle, WA 98109, United States; Howard Hughes Medical Institute, 1100 Fairview Ave N, Seattle, WA 98109, United States; Computational Biology Program, Fred Hutchinson Cancer Center, 1100 Fairview Ave N, Seattle, WA 98109, United States; Department of Statistics, University of Washington, 1410 NE Campus Pkwy, Seattle, WA 98195, United States; Howard Hughes Medical Institute, 1100 Fairview Ave N, Seattle, WA 98109, United States; Department of Genome Sciences, University of Washington, 3720 15th Ave NE, Seattle, WA 98195, United States

**Keywords:** SARS-CoV-2, escape, clonal interference, evolution, deep mutational scanning

## Abstract

SARS-CoV-2 has evolved increased resistance to human polyclonal antibody responses. But, how it escaped individual monoclonal antibodies from these responses has not been thoroughly explored. Cao et al. used deep mutational scanning to identify mutations that allow SARS-CoV-2 to escape individual antibodies, doing so for hundreds of different antibodies. Here, we use these data to reconstruct how the virus escaped each antibody in nature. For each antibody, we predict how levels of escape changed in the global SARS-CoV-2 population over time. For many antibodies, these levels dramatically fluctuated due to escape mutations being displaced by clade-turnover events. We validate predicted patterns using pseudovirus neutralization data. Fitness effects estimated from natural sequences suggest that mutations are displaced due to clonal interference between clades and that the order in which mutations arose is shaped by changing selective pressures. Overall, this work suggests that SARS-CoV-2 evaded polyclonal responses via complex evolutionary dynamics.

## Introduction

Severe acute respiratory syndrome coronavirus 2 (SARS-CoV-2) rapidly evolves, in part to escape human antibody-mediated immunity. In the first few years of the pandemic, researchers isolated hundreds of neutralizing monoclonal antibodies from humans (Hansen et al. 2020, Robbiani et al. 2020, Zost et al. 2020, Clark et al. 2021, Jones et al. 2021, Starr et al. 2021a, Cameroni et al. 2022, Cao et al. 2022a, 2022b, Park et al. 2022a, 2022b, Wang et al. 2022a, 2022b, Cao et al. 2023, Guenthoer et al. 2023). These antibodies target multiple epitopes on the spike protein, and several antibodies were approved as therapeutics. However, within a matter of years, the virus evolved resistance to nearly all of these antibodies (Cao et al. 2023, He et al. 2023). As the pandemic continues and human antibody responses are updated upon new exposures, the virus continues to evolve resistance (Jian et al. 2024), making it important to understand the evolutionary processes at play.

How SARS-CoV-2 evolved resistance to human antibody-mediated immunity during the first few years of the pandemic is one of the best-documented stories of immune escape to date, and some aspects of the story are very clear. In general, the virus’s resistance to human sera and monoclonal antibodies tended to increase over time upon the appearance of new variants of concern (Collier et al. 2021, McCallum et al. 2021, Cameroni et al. 2022, Liu et al. 2022, Planas et al. 2022, Cao et al. 2022a, 2022b, Addetia et al. 2023, Cao et al. 2023, He et al. 2023). And key mutations that drove escape were identified using a combination of bioinformatics and laboratory experiments (Rambaut et al. 2020, Aksamentov et al. 2021, Emma 2021, Wang et al. 2021, Greaney et al. 2021a, 2021b, Starr et al. 2021b, Ai et al. 2022, Chen et al. 2022, Liu et al. 2022, Cao et al. 2022a, 2022b, 2023, Cox et al. 2023, Dadonaite et al. 2023).

However, some aspects of this evolutionary story have not been fully resolved. For instance, while it is clear that the virus’s overall resistance to antibodies steadily increased, we still lack a complete story of how the virus evolved resistance to each of the large number of monoclonal antibodies that collectively made up the human polyclonal response. How did the virus navigate this complex immune fitness landscape in nature? Past studies have identified groups of antibodies that were escaped at different times by different mutations and variants of concern. However, these studies primarily focused on several well-studied antibodies, leaving room for a more comprehensive analysis.

Here, we sought to build on prior work by analyzing escape from hundreds of individual antibodies isolated from humans. Specifically, for each antibody, we sought to answer the following questions: When did the virus population escape the antibody? Did levels of escape in the viral population steadily increase over time, or were patterns more rugged? Which mutations drove escape? And how do patterns of escape relate to the frequent clade-displacement events in SARS-CoV-2’s evolution?

To answer these questions, we leveraged data from a series of studies by Cao et al. (2022a, 2022b, 2023), which used high-throughput experiments to isolate and characterize thousands of human monoclonal antibodies from the first few years of the pandemic. Specifically, they isolated antibodies that bound to the SARS-CoV-2 spike receptor-binding domain (RBD), which is a major target of neutralizing antibodies (Piccoli et al. 2020, Greaney et al. 2021a). For each antibody, they then used deep mutational scanning (DMS) to identify which mutations allow the Wuhan-Hu-1 RBD to escape antibody binding. They also measured the neutralization potency of these antibodies against pseudoviruses bearing spike proteins of several variants of concern, finding that the virus evolved resistance to nearly all antibodies with neutralizing activity. This dataset is unique among viruses in terms of its exceptional size, and thus offers a unique opportunity to separate a polyclonal response into many individual components and then study escape from each one.

For each of 1603 antibodies from Cao et al. we used the DMS data to estimate an antibody-escape score for each of thousands of globally circulating SARS-CoV-2 sequences from the first 3.5 years of the pandemic. For each antibody, we then computed an escape trajectory quantifying how the average escape score changed in the viral population over time. The trajectories follow several major patterns that differ in terms of their shape and the timing of escape. We validated these patterns using the neutralization data from Cao et al. and Wang et al. (Wang et al. 2022a). Some trajectories underwent large fluctuations over time as a result of clade-displacement events that reduced the frequency of escape mutations in the viral population. Fitness effects of mutations estimated from sequences in nature suggest that the displacement of escape mutations was often due to clonal interference. The fitness effects also suggest that changes in selective pressure impacted the order in which escape mutations arose.

## Methods

### Data and code availability

See https://github.com/matsengrp/ncov-ab-escape for input files, code, and key results files from our analysis. Key results files include:

- a file with metadata on the viral sequences from our analysis (https://github.com/matsengrp/ncov-ab-escape/tree/main/results/viral_metadata.csv).
- a file with site-level escape scores (https://github.com/matsengrp/ncov-ab-escape/tree/main/results/processed_input_data/escape.csv).
- a directory with files with fitness effects estimated from natural sequences (https://github.com/matsengrp/ncov-ab-escape/tree/main/results/aamut_fitness/).
- a file with each antibody’s escape trajectory and escape cluster (https://github.com/matsengrp/ncov-ab-escape/tree/main/results/trajectories.csv).

The README.md file in the repository’s root directory describes the repository’s contents in more detail.

### Input DMS data

We obtained the input DMS data from Cao et al. (2023). Their repository https://github.com/jianfcpku/convergent_RBD_evolution includes the DMS data for each antibody, as well as other metadata associated with each antibody, including the antibody’s source and its neutralization data against variants of concern. For practical reasons, the set of publicly available escape scores, which we use in this study, was filtered to remove data at sites where all escape scores were close to zero for a given antibody. We assign these filtered-out mutations escape scores of zero for that antibody.

Of the 3333 antibodies with data, we curated a set of 1603 using the following filters: (i) the antibody was strongly escaped in the DMS experiments, defined as the $e_{a,r,m}$ values for that antibody summing to $>2$ for at least one site, (ii) the antibody was isolated from a human exposed to SARS-CoV-2 (rather than SARS-CoV-1), and (iii) the antibody had pseudovirus neutralization data for all variants from the Cao et al. study. There were 2326 antibodies that passed the first filter, 1616 antibodies that passed the first two filters, and 1603 antibodies that passed all three filters.

### Sampling globally circulating viruses

We used a modified version of the ncov Nextstrain (Hadfield et al. 2018) workflow (https://github.com/nextstrain/ncov), to sample 3942 viral sequences from a set of about 100 000 publicly available sequences, each a consensus sequence from a SARS-CoV-2-infected individual. We sampled the viruses such that their collection dates were roughly uniform during the first 3.5 years of the pandemic, and such that their collection locations were roughly uniform among many regions around the globe to reduce regional bias. There were two reasons we did not use all available sequences. First, the 3942 sequences resulted in about 200 sequences for each 0.2-year sliding window, which was sufficient to accurately estimate population-averaged trends in each window. Second, the Nextstrain workflow involves building a maximum-likelihood phylogenetic tree of the sequences, which is not feasible with all available sequences. See https://github.com/matsengrp/ncov-ab-escape for the specific code and input files that we used to run this workflow, as well as key output files, including a file that lists for each viral sequence: the set of amino-acid mutations in that sequence’s spike protein relative to the Wuhan-Hu-1 reference strain, the year the sequence was sampled, and the Nextstrain clade associated with the sequence. The repository also includes the phylogenetic tree of these sequences.

### Computing viral escape scores and escape trajectories

We computed viral escape scores from site-level $x_{a,r}$ values using the approach described in the Results. We used the multiple-sequence alignment of spike sequences from above to define the set of mutations in each sequence relative to the Wuhan-Hu-1 reference sequence (called ‘Wuhan-Hu-1/2019’ in the alignment). We only considered mutations in the RBD, since the DMS data were specific to this domain. See https://github.com/matsengrp/ncov-ab-escape for the specific code we used to compute viral escape scores. We adapted this code from the escape calculator (https://github.com/jbloomlab/SARS2-RBD-escape-calc/) initially described in (Greaney et al. 2022).

When computing escape trajectories, we chose a sliding-window size of 0.2 years for two main reasons. First, it was small enough that we could clearly resolve large changes in mutational frequencies, which often tracked with clade-turnover events that occurred over several months. Second, it was big enough that we could accurately estimate population-averaged trends in each window using the 3942 viral sequences. Changing the window size by a factor of two did not substantially change the observed patterns in escape trajectories.

In Supplementary Fig. S9, we computed viral escape scores from mutation-level $e_{a,r,m}$ values using the following approach. For a given antibody $a$, we took the set of $e_{a,r,m}$ values for that antibody and normalized them so that they had a maximum value of 1.0. We did so by dividing each value in the set by the maximum value in the set. This gave a set of normalized mutation-level values ($n_{a,r,m}$). Next, we computed the escape score of each virus $v$ from each antibody as:


\begin{align*} & e_{a,v} = \min\left(1, \sum_{m}n_{a,r,m}\right) \end{align*}

where the sum is over all mutations in the virus’s RBD relative to the Wuhan-Hu-1 RBD used in the DMS experiments.

### Clustering of escape trajectories

We used *k*-means clustering to cluster trajectories into 20 groups. As input to the clustering algorithm, we used the high-dimensional vector of escape scores encoding each trajectory (one value for every 0.2 years in the sliding window over time).

### Analyzing escape scores

We used the COV CATNAP tool (Yoon et al. 2015) to explore the sea of literature on SARS-CoV-2 antibodies, helping us identify studies with neutralization data relevant to our work. We used the CoVariants website (https://covariants.org/) to identify mutations in variants of concern (Emma 2021).

### Estimating fitness effects of mutations at the level of clades

For this step, we used the previously published computational pipeline at https://github.com/neherlab/SARS2-mut-fitness-v2, which is described in detail in Haddox et al. (2025). We ran this pipeline using the same UShER tree described in Haddox et al. (from 24 April 2024), using a custom configuration file. See https://github.com/matsengrp/ncov-ab-escape for the custom configuration file that we used and for files reporting the estimated fitness effects.

As input, the above pipeline ingests mutational-counts data from the Bloom and Neher pipeline (Bloom and Neher 2023) at https://github.com/jbloomlab/SARS2-mut-fitness. These data come from taking the input tree, dividing it into separate clades, and then computing mutational counts for each clade. The Haddox et al. pipeline takes these clade-specific mutational counts and uses them to estimate clade-specific fitness effects. Specifically, for each amino acid mutation $m$ in a given clade, the pipeline estimates its fitness effect as: $f_{m} = \log ( (a_{m} + P) / (e_{m} + P) )$, where $a_{m}$ is the mutation’s actual count, $e_{m}$ is the mutation’s expected count, $P$ is a pseudocount of 0.5, and log is the natural log. Actual counts come directly from the counts data from the Bloom and Neher pipeline. Expected counts are derived from the log-linear neutral model described in Haddox et al.

### Estimating fitness effects of mutations in a sliding window over time

For this step, we used a new computational pipeline that we are publishing as part of this study. The code to run this pipeline is available at https://github.com/matsengrp/s2trajectory. See https://github.com/matsengrp/ncov-ab-escape for implementation details, and for an output file giving the actual and expected count of each mutation in each sliding window, which we used to compute fitness effects as described below.

This pipeline is similar to the clade-based pipeline from (Bloom and Neher 2023) and (Haddox et al. 2025) and involves the following steps. As input, the pipeline takes a UShER phylogenetic tree of SARS-CoV-2 sequences, along with associated metadata including the collection date, host, and assembled genome length of each sequence. We used the same tree as above. We filtered the tree to remove leaf nodes that: (i) did not have a precise collection date (known day, month, year) or had a collection date before 1 December 2019, (ii) were sampled from a non-human host, and (iii) had a genome assembly length outside of the range of 28 000 to 32 000 bp. We then used chronumental (Sanderson 2021) to estimate a time tree of the filtered sequences. As input, we used the filtered tree and the reported collection time of each leaf node. The output is a predicted time for both internal and leaf nodes. We verified that predicted and reported times were close for leaf nodes (data not shown).

Next, we computed the actual counts of mutations along the branches of the tree in a sliding window over time. We used a window with a width of 73 days (0.2 years) that slides along the tree with a step size of 36 days ( 0.1 years). For each window, we identified all subtrees in the window (Supplementary Fig. S10). Each subtree consists of a founder node, defined as the child node of a branch where the child node is between the window start time and end time, and the parent node is before the window start time. Each subtree also consists of all nodes that descend from the founder node and are also between the window start time and end time. We only consider subtrees that have at least two nodes (i.e. the founder node and at least one descendant node). For each subtree in a window, we made a list of all possible single-nucleotide codon mutations to the protein-coding genes in the founder node’s genome sequence. Then, for each mutation in this list, we recorded two things. First, we recorded the number of times the mutation was observed along branches in the subtree, giving the mutation’s actual count. Similar to (Bloom and Neher 2023), we ignored branches that: (i) had more than four nucleotide mutations, (ii) had more than one mutation i.e. a reversion to the Wuhan-Hu-1 reference sequence, (iii) had more than one mutation i.e. a reversion to the subtree founder sequence, (iv) had more than one mutation to the same codon. Second, we recorded the size of the subtree, defined as the total number of mutations observed along the subtree’s branches (ignoring the same set of branches as above). This number is the same for all mutations in a given subtree, and we use this number to quantify each mutation’s ‘evolutionary opportunity’. Next, for each mutation with data in the window, we summed the mutation’s actual count and evolutionary opportunity across all subtrees in the window.

Next, for each window, we estimated each mutation’s expected count, which quantifies how many times we would expect to observe the mutation if it had a neutral fitness effect. We did so by multiplying a mutation’s evolutionary opportunity in a given window by the mutation’s neutral rate. We used the neutral mutation rates tabulated in https://github.com/matsengrp/s2trajectory/blob/main/data/master_table_all_tree.csv. We derived these rates using the neutral mutation model described in (Haddox et al. 2025). In this model, rates depend on four features: (i) the mutation type (e.g. A$\rightarrow $C, A$\rightarrow $G, etc.; see the ‘mut_type’ column in the table), (ii) the genomic region of the mutated site (all sites in spike are within the same genomic region, with entries of False in the ‘nt_site_before_boundary’ column), iii) whether the mutated site is predicted to be base paired in the genome’s RNA structure (see the ‘unpaired’ column), (iv) the mutated site’s local sequence context, defined by identity of the nucleotides at site indices $n-1$ and $n+1$ for a mutated site with index $n$ (see the ‘motif’ column). Each row in the table corresponds to a unique set of the above four features. The model was trained to use these features to predict the actual counts of synonymous mutations along a set of filtered branches from the entire UShER tree. The column ‘predicted_count’ gives the prediction for a synonymous mutation type matching the features in a given row. We converted these counts to rates by dividing the counts by the total number of mutations along the branches in the training data (10 223 849 mutations). The column ‘rate’ reports the predicted neutral rate for each row. The synonymous mutations used in training are all at highly conserved sites, such that each mutation’s evolutionary opportunity is roughly equal to the total number of mutations from above. Thus, the rates are counts per unit of evolutionary opportunity. Multiplying a mutation’s rate by its evolutionary opportunity gives expected counts for that mutation under the neutral evolutionary model.

Finally, for each window, we estimated each mutation’s fitness effect. To do so, we first summed counts across all nucleotide mutations that encode the same amino acid mutation at the same site in the same gene. Then, we computed the fitness effect of each unique amino acid mutation $m$ in the window. We did so using the same equation as in the previous section. In Fig. 6, the plots only show the fitness effect for a given mutation in a given window if that mutation has $>5$ expected counts or $>5$ actual counts.

## Results

### Estimating levels of antibody escape over time

Of the $\sim $3000 antibodies characterized by Cao et al. we analyzed a set of 1603 antibodies that were filtered to only include ones isolated from humans exposed to SARS-CoV-2 (as opposed to SARS-CoV-1) and ones for which the DMS experiments detected strong escape mutations (see *Methods*). The antibodies were isolated from people with a variety of exposure histories, including vaccination with the wild-type (WT)-based vaccine, infection with WT variants, or WT vaccination followed by breakthrough infections with BA.1, BA.2, or BA.5 variants (Supplementary Table S1), providing a sizable sample of the human polyclonal antibody response during this window in time. As described more below, the antibodies target several epitopes and have a range of abilities to neutralize pseudoviruses bearing the D614G spike variant.

**Figure 1:**
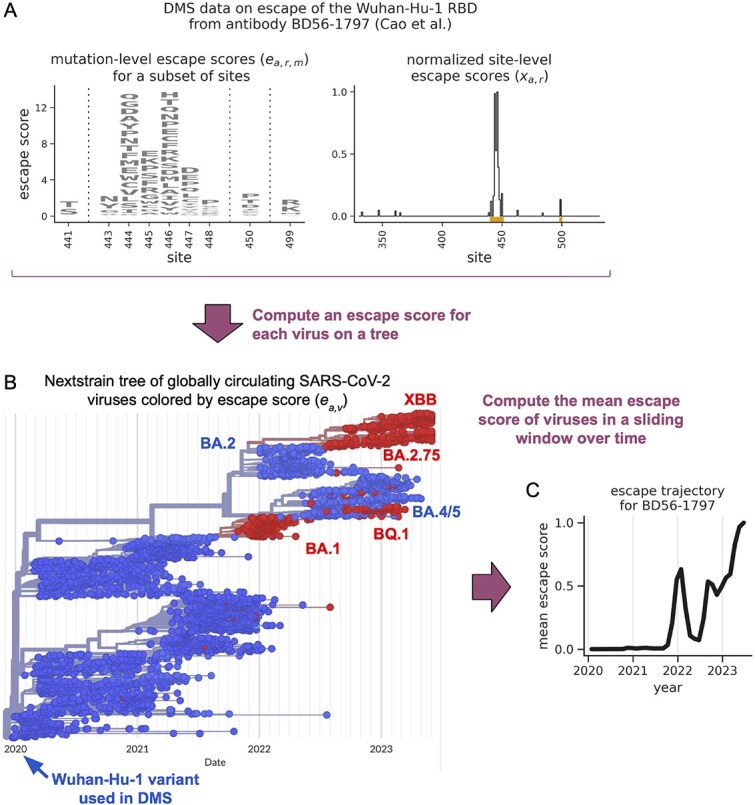
Strategy to estimate levels of antibody escape over time, illustrated for a single example antibody. (A) DMS data from Cao et al. quantifying the effects of mutations to the Wuhan-Hu-1 RBD on its ability to escape binding by an example antibody, BD56-1797. The left plot shows amino acid-level escape scores ($e_{a,r,m}$) for the subset of sites where scores sum to $>1$. The right plot shows the normalized site-level escape scores ($x_{a,r,}$), with orange rectangles identifying the sites shown in the left plot. (B) A time-resolved Nextstrain phylogenetic tree of all 3942 globally circulating SARS-CoV-2 viruses under analysis, coloured by each virus’s escape score ($e_{a,v}$) against antibody BD56-1797, as computed from $x_{a,r}$ values. The tree is coloured using a continuous blue-red colour scale that ranges from 0 to 1, with most scores being close to either 0 or 1. (C) A 0.2-year sliding-window average of the escape scores shown in panel B. This curve defines SARS-CoV-2’s ‘escape trajectory’ for antibody BD56-1797. We separately compute escape trajectories for each of the 1603 antibodies under analysis.

For each antibody, we sought to estimate how levels of antibody escape changed among globally circulating SARS-CoV-2 viruses over time. We did so using the following approach, illustrated for a single example antibody in Fig. 1. As input, we used the DMS data from Cao et al. For each antibody, these data measure the effects of thousands of amino-acid mutations spanning the length of the Wuhan-Hu-1 RBD on escape from antibody binding. Specifically, they displayed a mutant library of RBD variants on the surface of yeast cells (where each cell expresses a single RBD variant), incubated the cells with a given antibody, depleted antibody-bound cells from the library using magnetic-activated cell sorting, and then deeply sequenced the library before and after the depletion step to quantify how much each variant escaped antibody binding. From these data, they computed an escape score for each mutation, with scores ranging from 0 to 1, where 0 indicates that the mutation did not confer escape and 1 indicates that the mutation conferred strong escape. Figure 1A shows the distribution of escape scores for an example antibody. Also as input, we used Nextstrain (Hadfield et al. 2018) to obtain a set of 3942 SARS-CoV-2 genome sequences that were sampled from many countries around the globe and roughly uniformly over time during the first 3.5 years of the pandemic (Fig. 1B).

For each antibody, we used the DMS data to estimate an escape score for each of the 3942 viral sequences from above, doing so using a modified version of the strategy from (Greaney et al. 2022). Specifically, if $e_{a,r,m}$ is the escape score of the amino-acid mutation $m$ at site $r$ of the RBD against antibody $a$, we quantify how much mutating that site tends to escape the antibody as:


\begin{align*} & x_{a,r} = \frac{s_{a,r}}{\max_{r^{\prime}} s_{a, r^{\prime}}}, \text{where } s_{a,r} = \sum_{m}e_{a,r,m} \end{align*}

such that the maximum $x_{a,r}$ value across all sites is always equal to one for a given antibody. Next, we let $e_{a,v}$ quantify the escape score of virus $v$ from the antibody as:


\begin{align*} & e_{a,v} = \min\left(1, \sum_{r\in M_{v}}x_{a,r}\right) \end{align*}

where $M_{v}$ is the set of sites that are mutated in the virus’s RBD relative to the Wuhan-Hu-1 RBD used in the DMS experiments. We cap $e_{a,v}$ values at a maximum of 1, as values of 1 already indicate strong escape. Figure 1B shows virus-level escape scores for the example antibody mapped onto a time-resolved tree of the viruses.

The above approach uses site-level $x_{a,r}$ values to compute viral escape scores, but this isn’t the only possible choice. In principle, it would be more accurate to use mutation-level $e_{a,r,m}$ values since different amino acid mutations at the same site can have different effects. However, we chose the site-level approach because the mutation-level values are substantially noisy. As shown below, the site-level approach resulted in a better correlation between predicted viral escape scores and pseudovirus neutralization data, probably because site averaging corrects for the noise to some degree. Further, we show that our basic findings do not depend on the approach used.

Finally, for each antibody, we quantified how the average escape score in the viral population changed over time in a 0.2-year sliding window. This sliding-window average, illustrated in Fig. 1C for the example antibody, defines the virus’s ‘escape trajectory’ for a given antibody, and is a core part of our subsequent analyses.

We note that the Cao group has also published datasets that characterize antibodies elicited by infections later in the pandemic (Jian et al. 2024, Yisimayi et al. 2024). The reason why we do not include these datasets in our analysis is that the associated DMS experiments were performed in genetic backgrounds of more evolved RBD variants, rather than the Wuhan-Hu-1 RBD variant. By excluding these additional datasets, we ensure that all viral escape scores are relative to Wuhan-Hu-1.

### Antibody-escape trajectories follow several main patterns

**Figure 2:**
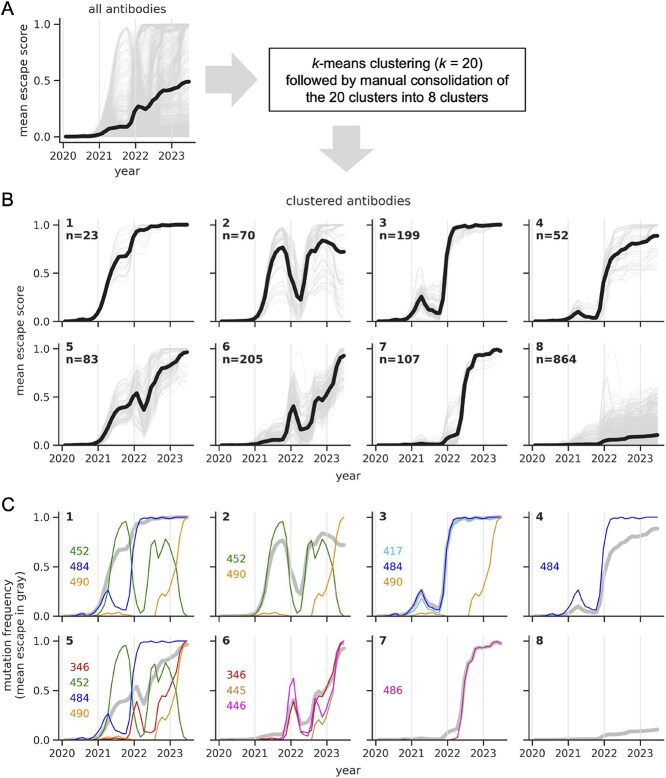
Clusters of antibody escape trajectories and the mutational dynamics underlying escape. (A) Average escape trajectory across all antibodies (bold black line) and trajectories for individual antibodies (light grey lines). (B) Escape trajectories partitioned into clusters, with the bold black line showing the cluster average and the light grey lines showing trajectories for individual antibodies. The top left corner of each plot reports the cluster label (1–8; clusters are roughly ordered according to when the virus escaped them) and the number of antibodies within each cluster. (C) For each cluster, the frequencies of mutations at sites that are key drivers of the escape trajectories (narrow colourful lines), along with the average escape trajectory for a given cluster, as in panel B (bold grey line). Mutational frequencies are computed using the same 0.2-year sliding windows as the trajectories.

As expected, when we averaged the escape trajectories across all 1603 antibodies, the resulting average trajectory showed a gradual increase in escape score over time, with the increase starting at about a year into the pandemic when the first variants of concern began to spread (Fig. 2A). The average trajectory only reaches a maximum value of $\sim $0.5, as a subset of antibodies are not predicted to be strongly escaped. This subset almost entirely consists of antibodies with little-to-no neutralizing activity, as described below.

In contrast, when we examined escape trajectories of individual antibodies, we saw a variety of trends. To identify these trends, we first clustered the trajectories into 20 clusters using *k*-means clustering, which revealed fine-grained trends (Supplementary Fig. S1). We then manually consolidated these 20 clusters into 8 clusters by combining clusters with similar escape patterns. These eight clusters highlight interesting biological patterns in the data (Fig. 2B). Average escape trajectories differ between clusters in a few key ways. First, they differ based on the timing of escape, with different clusters showing sequential waves of escape that span multiple years. Second, they differ in their shape. Some trajectories steadily increase over time before plateauing at a high escape score (e.g. clusters 1 and 7), while others show large fluctuations, where the escape score starts to increase, then substantially decreases before increasing to a high escape score (e.g. clusters 2, 3, 6). The trajectories in cluster 8 show no increase in escape score or plateau at values well below one. Individual trajectories are somewhat continuous between certain pairs of clusters (e.g. clusters 3 and 4) due to continuity in antibody DMS profiles (Cao et al. 2023).

In the next three sections, we examine the escape mutations that gave rise to these patterns, we examine the epitopes and neutralization activity of antibodies from each cluster, and we validate cluster-averaged escape patterns using experimental pseudovirus neutralization data.

### Some escape trajectories arose through complex mutational dynamics

For each cluster, we sought to identify key sites that drove escape. We did so by searching for sites that had an intermediate-to-high escape score ($x_{a,r}>0.4$) for at least a quarter of the antibodies in a given cluster, and where mutations reached a high frequency ($>0.9$) in nature. Figure 2C lists the sites that met these criteria for each cluster, and shows how changes in mutational frequencies at these sites drove each cluster’s average escape trajectory.

For some clusters, the average escape trajectory closely tracks with the frequency of mutations at a specific site. For instance, the average escape trajectory from cluster 7 closely tracks with the frequency of mutations at site 486, while the average escape trajectory from cluster 3 closely tracks with the frequency of mutations at either 417 or 484. The reason that the cluster 3 trajectory tracks with two different sites is that antibodies from this cluster tend to be strongly escaped by mutations at either 417 or 484 (Supplementary Fig. S2; compare cluster 3 epitopes A and C), and escape mutations at these sites are genetically linked, such that both groups of antibodies have similar escape trajectories.

In contrast, for other clusters, the average escape trajectory does not just track with the frequency of mutations at a single site, but is instead shaped by a combination of escape mutations at multiple sites in the same antibody epitope. Often, these escape mutations undergo large fluctuations over time, with one strong escape mutation declining in frequency while another rises, such that escape is maintained over time by different mutations (see clusters 1, 2, and 5).

Some sites are key drivers of escape for multiple clusters. This results from antibodies from different clusters having overlapping, but distinct epitopes (Supplementary Fig. S2). For instance, clusters 1 and 2 share two key sites where mutations drive escape (sites 452 and 490), but differ in a single key site (484). Similar patterns are apparent for clusters 1 and 3 and clusters 5 and 6. These subtle differences in antibody epitopes give rise to large differences in escape trajectories.

### Antibody epitopes and neutralization potencies

**Figure 3:**
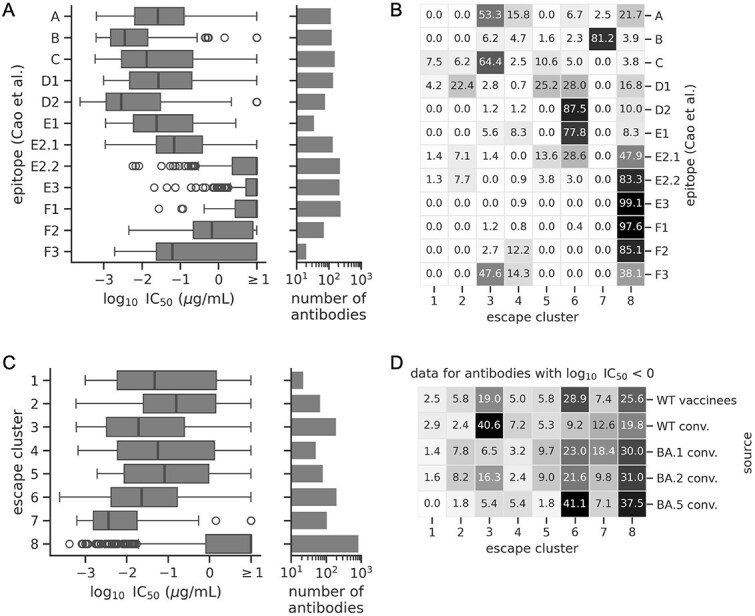
Antibody epitopes and neutralization potencies. (A) Data for the 1603 antibodies grouped by epitope (defined by Cao et al.), with the left plot showing the distribution of $\log _{10}\mathrm{IC}_{50}$ values quantifying the ability of an antibody to neutralize pseudoviruses bearing the D614G spike, and the right plot showing the number of antibodies within each epitope. (B) A heat map showing the percent of antibodies in a given epitope that are in a given escape cluster (Fig. 2B), such that each row sums to 100. (C) Same as panel A, but showing data for antibodies clustered by escape trajectory. (D) A heat map showing the percent of antibodies from a given source that are in a given escape cluster, such that each row sums to 100 (conv. = convalescent). The plot only shows data for antibodies with appreciable neutralizing activity against the D614G variant ($\log _{10}\mathrm{IC}_{50} < 0$$\mu $g/ml).

The antibodies in our analysis target several epitopes, defined by Cao et al. based on clustering of their DMS profiles. All of these antibodies bind the RBD, but they have a range of abilities to neutralize pseudoviruses bearing the original Wuhan-Hu-1 D614G spike variant (Fig. 3A). Antibodies with lower neutralization potency may be weaker binders, or they may bind in such a way i.e. less disruptive to viral entry into cells. As might be expected, antibodies that target the same epitope often fall in the same escape cluster, but not always, such that clustering trajectories by epitope conflates certain escape patterns (Fig. 3B; Supplementary Fig. S3). Sometimes, antibodies targeting *different* epitopes fall in the same cluster, either due to escape mutations in different epitopes being genetically linked or, in the case of cluster 8, due to little-to-no predicted escape.

The antibodies from clusters 1 through 7, which are predicted to be strongly escaped, often neutralize the D614G variant, while those from cluster 8, which are not predicted to be strongly escaped, tend to have little-to-no neutralizing activity (Fig. 3C). This pattern is consistent with the expectation that neutralizing antibodies exerted the greatest selection on the virus.

However, the antibodies escaped first were not always the most potent. Antibodies from clusters 3, 6, and 7 stand out as having especially high neutralization potency and abundance (Fig. 3C). Cluster 3 antibodies are predicted to be among the first that were completely escaped, as defined by trajectories reaching values close to one (Fig. 2B). However, cluster 6 and 7 antibodies were among the last to be completely escaped, raising the question of why these antibodies were not escaped sooner.

One hypothesis is that antibody abundance changed over time. Grouping antibodies by host source shows that neutralizing antibodies from WT exposures, which occurred earlier in the pandemic, are particularly enriched in cluster 3, while those from the BA.5 infections, which occurred later, are particularly enriched in cluster 6 (Fig. 3D). Thus, shifts in immunity may partially explain the order of escape. However, aside from clusters 3 and 6, the distribution of neutralizing antibodies between clusters is largely similar between host sources (Fig. 3D), likely due to imprinting effects described by Cao et al. (2023). Thus, factors other than antibody potency and abundance may have contributed to the order of escape.

### Validation of average escape patterns from each cluster using pseudovirus neutralization data

We next sought to validate cluster-averaged patterns. For neutralizing antibodies in our analysis, we hypothesized that predicted viral escape scores would correlate with experimentally measured neutralization data that are available for several variants of concern. These data come from Cao et al. and Wang et al. (Wang et al. 2022a). For each antibody from Cao et al. the authors measured the ability of the antibody to neutralize pseudoviruses bearing one of several spike variants, including the D614G variant and several Omicron variants of concern. For a small subset of these antibodies, Wang et al. used the same approach to measure the ability of each antibody to neutralize the D614G variant, several pre-Omicron variants of concern, and BA.1. Together, these spike variants are widely distributed across the tree of viruses in our analysis, are founder sequences of large clades, and capture large changes in predicted escape scores, making them well-suited for validating predicted trends.

**Figure 4:**
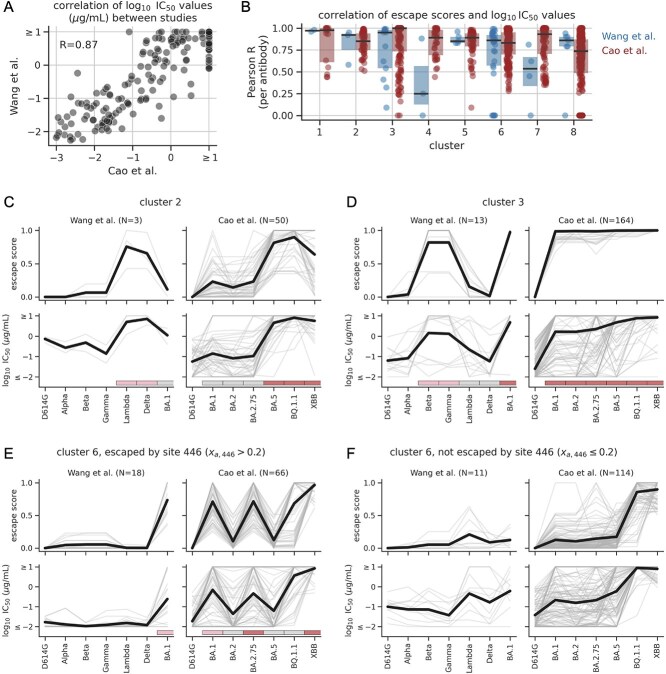
Predicted escape scores correlate with pseudovirus neutralization data. (A) Correlation of $\log _{10}\mathrm{IC}_{50}$ values between studies. (B) Distributions of per-antibody Pearson correlation coefficients quantifying the agreement between predicted escape scores and $\log _{10}\mathrm{IC}_{50}$ values for spike variants from a given study. Each dot corresponds to a single antibody and antibodies are grouped by cluster. Shaded boxes show interquartile ranges, and horizontal black lines show medians. We applied a floor of zero to each distribution. (C)–(F) Comparison of escape scores and $\log _{10}\mathrm{IC}_{50}$ values for antibodies from a given cluster. In each panel, the top row of plots shows escape scores and the bottom row shows $\log _{10}\mathrm{IC}_{50}$ values; the left column of plots shows antibodies with neutralization data from Wang et al. the right column shows antibodies with neutralization data from Cao et al. and N indicates the number of antibodies with data. The x-axis shows the spike variants assayed in each study, with variants ordered roughly chronologically by when they emerged in nature. Bold black lines show average values, while thin grey lines show values for individual antibodies. The $\log _{10}\mathrm{IC}_{50}$ values have an upper limit of detection of 1, and we floored the values at a lower limit of $-2$. Panels E and F show data for two groups of antibodies in cluster 6: ones escaped by mutations at site 446 ($x_{a,446}>0.2$) and ones that are not ($x_{a,446}\leq 0.2$). In panels C–E, the pink, grey, and red rectangles below the neutralization data correspond to the cluster-specific clade groupings from Fig. 5. Supplementary Figure S4 shows similar plots for the remaining clusters.

The above data were collected using large-scale neutralization assays, and as such, have a non-trivial level of noise. We quantified this noise by examining the correlation of $\log _{10}\mathrm{IC}_{50}$ values between Cao et al. and Wang et al. for antibody-variant combinations measured in both studies. These values were correlated (Pearson $R$ = 0.87), but often differ by one or more $\log _{10}$ units (Fig. 4A).

We found that the neutralization data are consistent with many of the cluster-averaged patterns of escape. We specifically focused on antibodies with appreciable neutralizing activity against the D614G variant ($\log _{10}\mathrm{IC}_{50} < 0$  $\mu $g/ml), since the neutralization data are only suitable for validating escape from neutralizing antibodies. Figure 4C–F show data for the three clusters with the largest fluctuations in average escape trajectories (clusters 2, 3, and 6). The predicted escape scores show striking increases and decreases between adjacent spike variants when the variants are ordered roughly by when they emerged. When averaging over all antibodies in a cluster, the changes in escape score are mirrored by changes in $\log _{10}\mathrm{IC}_{50}$ values (the bold black lines are similar between the corresponding upper and lower panels). At the same time, for a subset of individual antibodies, the changes are clearly not mirrored, either due to inaccurate predictions, experimental noise, or experimental limits of detection.

The antibodies from cluster 6 show two distinct neutralization profiles depending on whether the antibody is strongly escaped by mutations at site 446 or mutations at another site, most commonly site 346 (Fig. 4E and F, respectively). The key difference is that mutations at site 446 are present in the BA.1 and BA.2.75 variants, while mutations at site 346 are not. These two groups of antibodies still have similar predicted escape trajectories because mutations at site 346 reach high frequencies within the BA.1 and BA.2.75 clades, even though they are not present in the clade-founder sequences used in the neutralization assays (we describe this pattern in more detail below).

To quantify levels of agreement, for each antibody, we computed the Pearson correlation coefficient between escape scores and $\log _{10}\mathrm{IC}_{50}$ values among all spike variants from a given study (Fig. 4B). For each cluster of antibodies, the resulting median correlation coefficient was often between 0.75 and 1.0, with only a small subset of individual antibodies reaching low correlation coefficients. The median correlation coefficient for the Wang et al. study was low for cluster 4 and 7, though these medians are only derived from a total of 3 and 4 antibodies, respectively, and the average trajectories of these clusters, which are largely shaped by Omicron variants, are supported by the Cao et al. data (Supplementary Fig. S4). Unfortunately, most clusters only had a few antibodies with validation data from Wang et al. limiting our ability to robustly validate all trends using these data. But, overall, the data are still consistent with many of the cluster-averaged escape patterns.

One reason that the above correlation coefficients are high is that both escape score and neutralization resistance tend to generally increase with sequence divergence, regardless of cluster. To quantify this effect, we recomputed the correlation coefficients using the number of RBD mutations in a given variant in place of its escape score. For clusters 1 through 3 and 5 through 7, the resulting coefficients are often substantially lower than before for at least one study (Supplementary Fig. S5), indicating that escape scores capture more than just sequence divergence.

The validation data are difficult to interpret for cluster 8 antibodies. Most antibodies from cluster 8 are *non*-neutralizing, and so were not included in this neutralization-based validation analysis. For the subset of antibodies in this cluster that are neutralizing, we did not find evidence that escape scores capture more than sequence divergence (Supplementary Fig. S5). Further, since most escape scores from this cluster only slightly increase over time, a concern is that the increase is merely due to the summed effects of noise from the DMS experiment, such that the correlation with the neutralization data is artificial. Despite the modest increases in predicted escape score, there is a large increase in average $\log _{10}\mathrm{IC}_{50}$ values between the D614G variant and the BQ.1.1 and XBB variants (Supplementary Fig. S4), indicating that the DMS experiments may not have identified relevant escape mutations, possibly due to experimental noise, or possibly because the relevant mutations do not confer escape in the Wuhan-Hu-1 background.

### Large dips in escape trajectories track with clade-displacement events

**Figure 5:**
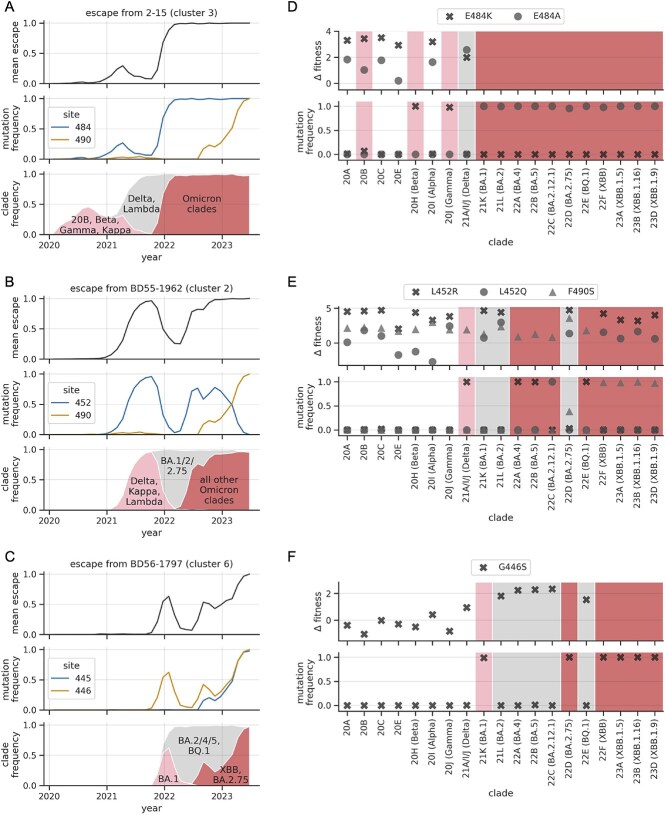
Clade-displacement events cause large dips in trajectories. (A)–(C) Each panel shows data for an example antibody from a given cluster. For each antibody, rows show the escape trajectory for that antibody, the frequency of mutations at key sites of escape (i.e. sites where $x_{a,r}>0.4$ and where mutations reach a frequency of $>0.9$), and the frequencies of groups of clades involved in displacement events that shape a given trajectory. Clades in the pink and red groups have escape mutations at appreciable frequencies, while clades in the grey group do not. Trajectories dip when grey clades displace pink clades and rise when red clades displace grey clades. We use Nextstrain clade definitions and compute frequencies using the same 0.2-year sliding windows used to compute the trajectories. (D)–(F) Each panel shows data for specific amino-acid mutations that drove escape trajectories in panels A–C (compare panels in the same row). In each panel, the bottom plot shows mutation frequencies in each of the indicated Nextstrain clades (with parenthetical names giving the variant of concern that corresponds to a clade’s founder sequence, when applicable). The frequency of these mutations within individual clades is either close to zero or one. For simplicity, we only show data for clades that reached $>7$% frequency. Clades are coloured according to how they are grouped in panels A–C. The top plot shows estimates of mutation fitness effects in each clade (effects are not estimated for clades that already have a mutation at a given site).

Clusters 2, 3, and 6 show large dips in escape trajectories (Fig. 2). As demonstrated above, these dips happen as a result of escape mutations decreasing in frequency in the viral population. These decreases are due to clade-displacement events. Figure 5A–C illustrates this phenomenon in the context of a single example antibody from each cluster for three clusters (other clusters can be found in Supplementary Fig. S6). In each case, the antibody escape trajectory begins to increase as an escape mutation rises in frequency in the viral population. The mutation is initially found in the group of clades coloured in pink in the figure. Next, the grey group of clades, which lack the mutation, displace the pink group. This causes a dramatic decrease in the overall frequency of the escape mutation, and thus the escape trajectory. Finally, the red group of clades, which have escape mutations, displace the grey group, causing the frequency of these mutations and the trajectory to increase again. (Observe the concordance between the ‘mean escape’ trajectory and the union of the pink and red clade frequency trajectories.) We see similar patterns with other escape mutations associated with fluctuating trajectories (Supplementary Fig. S6A and B).

The trajectories closely track with clade-displacement events because the frequency of escape mutations within individual clades is often either close to zero or one (see the bottom plot of each panel of Fig. 5D–F; the upper plot will be described in the next section). An exception to this trend is site 346, where escape mutations have intermediate frequencies in multiple clades (Supplementary Fig. S6E).

In the examples from Fig. 5, the clades that caused the trajectory of one antibody to decrease often caused the trajectory of another to increase. The spread of Delta and Lambda caused the cluster 3 antibody trajectory to decrease, but caused the cluster 2 antibody trajectory to increase. The spread of BA.1 caused the cluster 2 antibody trajectory to decrease, but caused the cluster 3 and cluster 6 antibody trajectories to increase. Such patterns suggest that clade-displacement events resulted in large trade-offs in resistance to different clusters of antibodies, despite the fact that the virus’s overall antibody resistance steadily increased over time.

### Evidence that clonal interference caused the displacement of escape mutations

We next sought to investigate why clade-displacement events were effective at driving down the frequencies of escape mutations in the viral population. We found this pattern intriguing, since SARS-CoV-2 can undergo genetic recombination (Turakhia et al. 2022), which provides a mechanism to consolidate beneficial mutations from different genetic backgrounds (Tamura et al. 2023). In each example from Fig. 5A–C and Supplementary Figs S6A and B, recombination would have offered a mechanism to introduce the escape mutation from the pink clades into the genetic background of the grey clades, helping to prevent the escape mutation from being displaced. According to our additive model, such recombination events would have been beneficial, as they would have increased escape scores of viruses in the grey clades. *De novo* mutation offers another mechanism by which escape mutations could have arisen and spread in grey clades. But, ultimately, none of the displaced mutations reached appreciable frequencies in the grey clades (Fig. 5D–F; Supplementary Fig. S6D–E).

There are multiple possible explanations for this pattern. One explanation is clonal interference. For organisms that do not undergo genetic recombination, clonal lineages with different beneficial mutations can compete with one another, delaying the rate at which beneficial mutations are fixed in the population (Crow and Kimura 1965, Hegreness et al. 2006, Kao and Sherlock 2008, Lang et al. 2013). Although SARS-CoV-2 does recombine, it is possible that recombination has not always been efficient enough to prevent competition between clades from displacing beneficial mutations. Likewise, the rate at which these mutations arise *de novo* and spread within a clade may not always have been high enough to prevent their displacement.

Another explanation is differences in selective pressures between clades. The escape mutations in the above examples may have had beneficial fitness effects in the pink clades, but neutral or deleterious effects in the grey clades. This could happen due to epistasis (the mutation is incompatible in the genetic backgrounds of the grey clades) or due to changes in external selective pressures (human humoral immune responses can change over time due to factors like exposure history and waning immunity), such that even if the mutation was introduced into a grey clade, it would not have conferred a selective advantage.

To investigate these possibilities, we used a previously published computational pipeline to estimate fitness effects of mutations from natural sequences (Bloom and Neher 2023, Haddox et al. 2025). As input, we used a phylogenetic tree of $\sim $16 million SARS-CoV-2 genome sequences. The pipeline estimates a mutation’s effect based on the number of times the mutation was observed to arise *de novo* on the branches of the tree versus the number of times it was expected to have arisen under neutral evolution. Specifically, it estimates the effect as the natural log of the ratio of observed counts to expected counts, such that mutations that arose more than expected have positive (i.e. beneficial) fitness effects, and vice versa for mutations that arose less than expected. It performs this estimation using a Bayesian approach that integrates over uncertainty in the observed and expected counts. A mutation’s effect is defined as the mean of the posterior probability distribution over all possible effects. The standard deviation of this distribution can be used to quantify the level of uncertainty associated with a given effect. We used this approach to estimate mutational effects at the level of individual clades in our analysis.

Under the clonal-interference hypothesis, we would expect escape mutations from the pink clades to have positive fitness effects in the grey clades that displace them. Indeed, we see this pattern for four out of the six displaced escape mutations. For instance, consider the G446S mutation plotted in Fig. 5F. This mutation was fixed in the pink BA.1 clade (see the bottom plot). The founder sequences of the grey clades (BA.2, BA.4, etc.) do not have this mutation. In each of the grey clades, the estimated fitness effect for G446S was a large positive number (see the top plot), indicating it was beneficial, occurring along branches of phylogenetic trees of these clades much more than expected under neutral evolution. These large positive effects are much larger than associated levels of uncertainty (each effect is $>2$-fold larger than the standard deviation of the corresponding posterior distribution; Supplementary Fig. S7C). Although G446S was observed to occur many times along the grey-clade trees, its overall frequency in the grey clades was still low. Mutations that arose many times may still have low overall frequencies if those mutations have not yet had time to increase in frequency. One way G446S could have been introduced into viral genomes from grey clades is via recombination with BA.1. The estimated fitness effects suggest such an introduction would have been beneficial. Yet, G446S never reached appreciable frequencies in the grey clades. Thus, these results suggest that neither recombination nor *de novo* mutation were efficient enough to prevent the large dip in frequency of the G446S mutation when BA.1 was replaced by the grey clades, pointing to clonal interference.

We see similar evidence of clonal interference for E484K (Fig. 5D; see the large positive effect of this mutation in the Delta clade), L452R (Fig. 5E; see the large positive effect of this mutation in the BA.1, BA.2, and BA.2.75 clades), and R346K (Supplementary Fig. S6E; see the large positive effect of this mutation in the BA.2 clade). As above, these large positive effects are much larger than associated levels of uncertainty (Supplementary Fig. S7). For R346K, we also find support for changing selective pressures between clades: the mutation is present at high frequency in the pink BA.1 clade and has a large positive fitness effect in the grey BA.2 clade (suggesting clonal interference), but has more modest positive effects in subsequent grey clades (suggesting changing selective pressures).

The remaining two displaced escape mutations (K417N and K417T) show little or no evidence of clonal interference. Each mutation has a roughly neutral fitness effect in the grey Delta clade that displaced it (Supplementary Fig. S6D; Supplementary Fig. S7D).

**Figure 6:**
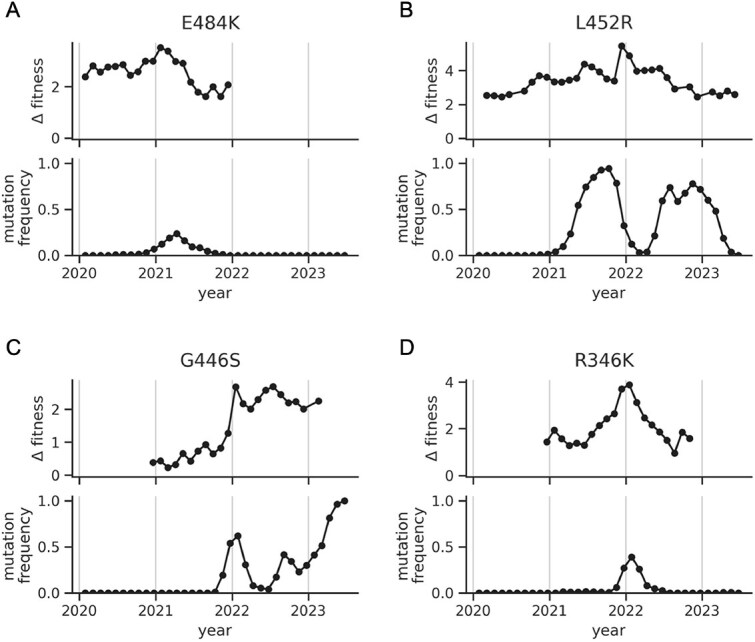
Fitness effects of mutations in a sliding window over time. Each panel shows data for a mutation that showed signs of clonal interference. In each panel, the bottom plot shows the mutation’s frequency in the sampled viruses in a sliding window of 0.2 years. The top plot shows the mutation’s estimated fitness effect, also in a sliding window of 0.2 years. In each case, when a mutation dips in frequency, it has a large positive fitness effect.

To complement the above clade-based analysis, we also estimated fitness effects of mutations in a 0.2-year sliding window over time, which offered better temporal resolution of these effects. We focused on the four mutations that showed signs of clonal interference from the clade-based analysis. In each case, in windows where the mutation is decreasing in frequency, its fitness effect is a large positive number (Fig. 6). This result indicates that the mutations were beneficial at the time of their displacement in context of sequences that did not already have that mutation. Thus, both the clade-based and time-based analysis are consistent with the clonal-interference hypothesis. Both analyses also showed evidence of changing selection over time. In a few cases, especially R346K, the mutation’s fitness effect is decreasing at the same time its frequency is decreasing. But, the fitness effects in these windows are still large and positive, suggesting that changes in fitness effects over time are not sufficient to explain the large dips in mutational frequencies.

### Shifts in mutational effects impact the order in which escape mutations arose

The key sites of escape from Fig. 2C can be divided into two broad groups based on whether or not escape mutations at a site reached appreciable frequencies before the emergence of Omicron in late 2021. They did at sites 417, 452, and 484, and did not at sites 346, 446, 486, and 490. Why did some escape mutations not appear before the emergence of Omicron?

The fitness data from the previous section indicate that the effects of some mutations shifted to become more beneficial upon the emergence of Omicron. We see this pattern for multiple key escape mutations at the second group of sites: R346T, G446S, and F486V [Fig. 5F; Supplementary Fig. S7E and F; compare fitness values in pre-Omicron clades (20A–21J) and Omicron clades (21K–23D)]. These positive shifts in fitness are greater than associated levels of uncertainty (Supplementary Fig. S7). Thus, these shifts could help explain why these three antibody-escape mutations only arose after the emergence of Omicron.

The above shifts could be due to multiple factors, including shifts in human immunity over time or epistasis with pre-existing mutations in Omicron clades. Consistent with the epistasis hypothesis, past work has identified multiple examples of mutations with enhanced antibody-escape effects in Omicron backgrounds (Starr et al. 2022b, Witte et al. 2023), including mutations at sites 346, 446, and 486. Such enhancement could explain the increased fitness of the above mutations in Omicron clades. Past work has also identified examples of escape mutations, such as G446S, that are deleterious to RBD-ACE2 binding in context of the Wuhan-Hu-1 RBD, but are buffered by other affinity-enhancing mutations in Omicron BA.1 (Moulana et al. 2022, Starr et al. 2022a). When we examined ACE2-binding effects of each of the key antibody-escape mutations from our analysis, we found that mutations at 417, 446, and 486 tended to have deleterious effects (Supplementary Fig. S8). If variants from Omicron clades are generally better at buffering such deleterious effects, that could also explain the increased fitness of G446S and F486V in Omicron. Further, while G446S is deleterious to ACE2 binding in pre-Omicron variants, its effect is much less deleterious in the BA.2 variant (Supplementary Fig. S8), indicating a reduced need for buffering in this Omicron variant.

In all, the above patterns suggest that there are multiple factors that can determine how quickly antibody-escape mutations appear and then fix in the viral population, including both clonal interference and changes in underlying selective pressures over time.

## Discussion

Leveraging the DMS data from Cao et al. we predicted antibody-escape scores for thousands of globally circulating viral variants, doing so for each of 1603 antibodies. For each antibody, we then quantified how the average escape score changes in the viral population over time. We distilled the resulting trajectories into eight major clusters. Although some trajectories steadily increase in escape score over time, others show striking fluctuations. We validated cluster-averaged trajectories by showing that predicted escape scores correlate with pseudovirus neutralization data for several variants of concern (using neutralization data from Cao et al. and Wang et al.). We showed that fluctuations in trajectories track with clade-displacement events that displaced key escape mutations from the population. Phylogenetically estimated fitness effects suggest that the mutations were often displaced due to clonal interference. The fitness estimates also indicate that the order in which escape mutations arose is shaped by changing selective pressures.

Previous studies have investigated the causes and consequences of SARS-CoV-2 antigenic evolution. Its evolution largely proceeds through clade-turnover events, with newer clades tending to be more resistant to human humoral immunity. Further, human immunity shifts over time, due to exposure history and waning immunity, and these shifts can help explain clade-turnover events (Meijers et al. 2023, Alexia Raharinirina et al. 2025). Our study builds on this work by helping answer two key questions, as considered in the first 3.5 years of the pandemic. First, while newer clades tended to be more resistant to polyclonal responses, how did their resistance change over time with respect to the many individual monoclonal antibodies that make up polyclonal responses? Second, what were the evolutionary forces that drove the observed patterns of monoclonal-antibody escape?

Our study helps answer the first question by examining a much larger number of antibodies than most previous studies. This allowed us to more comprehensively characterize patterns of escape and how many antibodies followed each pattern. For instance, past studies (He et al. 2022, Huang et al. 2022, Yuan et al. 2022, Cox et al. 2023, He et al. 2023) have also identified individual antibodies for which levels of resistance fluctuated between variants of concern over time, including antibodies with neutralization profiles that are similar to those of clusters 2, 3, and 6 (Supplementary Table S2). Building on these studies, our work indicates that a substantial fraction of the hundreds of neutralizing antibodies from our analysis have fluctuating escape trajectories. Thus, although our estimates indicate that the virus’s average antibody resistance tended to steadily increase over time, they also indicate that the underlying escape patterns were complex, with large trade-offs in resistance between different groups of antibodies.

For the second question, we uncover evidence that the observed patterns of escape were shaped by both clonal interference and changing selective pressures over time. Specifically, we find evidence that clonal interference has delayed the fixation of antibody-escape mutations in SARS-CoV-2’s evolution. This phenomenon has been observed for viruses that do not genetically recombine (Miralles et al. 1999, Strelkowa and Lässig 2012, Xue et al. 2017). However, it has been unclear whether it impacts SARS-CoV-2. Recombination has been detected among natural SARS-CoV-2 isolates (Turakhia et al. 2022), including an event that gave rise to the XBB variant of concern (Tamura et al. 2023), which spread globally. Thus, SARS-CoV-2 recombination can produce highly fit variants. Building on this work, our findings indicate that although SARS-CoV-2 recombination is common, it has not always been efficient enough to prevent beneficial escape mutations from being displaced, leading to clonal interference. In each example of this pattern that we characterized, the displaced mutation (or another at the same site) eventually rebounded in frequency after about six months to a year, suggesting that although clonal interference may have delayed SARS-CoV-2’s antigenic evolution, the delay was not long.

Our work emphasizes that it can take a substantial amount of time for positively selected mutations to reach appreciable frequencies in the global population. In examining clade-specific mutational fitness effects, we identified several instances where the *de novo* substitution rate of an escape mutation within a clade was $\sim $7–150 times higher than expected under neutral evolution, indicative of positive selection. But, in most such instances, the overall frequency of the mutation in the clade was still close to zero. Thus, high levels of positive selection do not necessarily lead to large increases in mutation frequency during the lifetime of a clade. This could help explain why recombination does not always prevent clonal interference: even if recombination produces fit variants at a low basal rate, those variants are not guaranteed to immediately become widespread.

Our work also adds support to the hypothesis that changing selective pressures impacted the order in which escape mutations arose. Previous studies have provided compelling experimental evidence that compensatory mutations in the BA.1 genetic background help buffer functionally deleterious effects of other BA.1 mutations that confer antibody escape (Starr et al. 2022a, Moulana et al. 2022). Studies have also shown that some mutational effects on antibody escape are potentiated in Omicron backgrounds (Starr et al. 2022b, Witte et al. 2023). Here, we add to this work by showing that fitness effects estimated from natural sequences show dramatic increases in Omicron clades compared to pre-Omicron clades for three key escape mutations from our analysis. And we show how these shifts can help rationalize the order in which key escape mutations from our analysis arose in nature.

Our study has several limitations. First, the DMS data measure the effects of mutations on RBD escape from antibody binding in a yeast-display system, not effects on escape from antibody neutralization in a viral context. This could help explain some of the discrepancy between the predicted escape scores and the experimental neutralization data. Second, the additive approach to predict antibody-escape scores makes multiple assumptions. It assumes no epistasis between mutations. It also assumes that all mutations at a site have the same effects, since it uses site-level $x_{r,a}$ values rather than mutation-level $e_{a,r,m}$ values. Despite these limitations, the predicted escape scores still correlate well with experimental neutralization data. Further, this correlation was actually higher when computing escape scores using site-level values rather than mutation-level values (Supplementary Fig. S9A). This is probably because site averaging reduces noise, as well as empirically resulting in increased levels of predicted escape (Supplementary Fig. S9B). The escape trajectories showed qualitatively similar patterns regardless of whether we used site-level or mutation-level values (Supplementary Fig. S9C). Similar DMS-based additive approaches have also been successful at predicting antibody escape for viral entry proteins from Influenza virus and Lassa virus (Carr et al. 2024, Welsh et al. 2024). Third, we assume that the set of 1603 antibodies we analyzed are broadly representative of human humoral immunity to the RBD. In support of this assumption, many of these antibodies target the same set of immunodominant epitopes identified by other studies (Hastie et al. 2021, Yuan and Wilson 2025). Fourth, the mutational fitness effects estimated from naturally occurring sequences are difficult to experimentally validate, and the trends indicating clonal interference and changing selective pressures are mainly supported by four and three mutations, respectively. We will be curious to see if similar trends are apparent in the ongoing evolution of SARS-CoV-2.

Overall, our work helps dissect how SARS-CoV-2 evaded the individual components of a polyclonal antibody response in nature. In the future, our analysis framework could be applied to other contexts. DMS is increasingly being used to identify mutations to viral entry proteins that confer antibody escape, both for SARS-CoV-2 (Alcantara et al. 2023, Dadonaite et al. 2024b, Jian et al. 2024, Lei et al. 2024) and for other viruses (Kikawa et al. 2023, Radford et al. 2023, Dadonaite et al. 2024a, Aditham et al. 2024, Carr et al. 2024, Larsen et al. 2024, Welsh et al. 2024). Further, many viruses have pre-existing Nextstrain (Hadfield et al. 2018) pipelines for creating curated multiple-sequence alignments from real-time surveillance data (https://nextstrain.org/). Our framework, and others like it, offers an exciting way to combine these sources of data to interpret patterns of viral evolution in nature.

## Supplementary Material

si_veaf104
