# Correction to: Clonal interference and changing selective pressures shape the escape of SARS-CoV-2 from hundreds of antibodies

**DOI:** 10.1093/ve/veag056

**Published:** 2026-08-14

This is a correction to: Hugh K Haddox, Omar Abdel Aziz, Jared G Galloway, Javen Kent, Cameron R Cooper, Chris Jennings-Shaffer, Will Dumm, Seth D Temple, Jesse D Bloom, Frederick A Matsen, Clonal interference and changing selective pressures shape the escape of SARS-CoV-2 from hundreds of antibodies, *Virus Evolution*, Volume 12, Issue 1, 2026, veaf104, https://doi.org/10.1093/ve/veaf104

In the originally published version of this manuscript, the Supplementary Material was mistakenly provided in .tex format. This .tex file has been replaced with a .pdf. No other changes have been made to the Supplementary Material.

