# Supplementary material for: Clonal interference and changing selective pressures shape the escape of SARS-CoV-2 from hundreds of antibodies
Source: Virus Evol. 2026 Feb 4;12(1):veaf104. doi: 10.1093/ve/veaf104 (PMC12936870; doi:10.1093/ve/veaf104)
Supplement: Supplementary file 1 [file si_veaf104.pdf]

Supplementary Information for: Clonal interference and changing selective pressures shape the escape of SARS-CoV-2 from hundreds of antibodies

Hugh K. Haddox, Omar Abdel Aziz, Jared G. Galloway, Javen Kent, Cameron R. Cooper, Chris Jennings-Shaffer, Will Dumm, Seth D. Temple, Jesse D. Bloom, Frederick A. Matsen IV

December 8, 2025

Figures and Tables

source	number of antibodies
WT vaccinees	205
WT convalescents	321
BA.1 convalescents	462
BA.2 convalescents	470
BA.5 convalescents	145

Table S1: Number of antibodies per source. Cao et al. isolated antibodies from humans with a variety of exposure histories. For the 1,603 antibodies from our analysis, the table shows how many antibodies were isolated from a human with a given exposure history.

tab:sources

antibody	similar to cluster	site(s) of escape
R1-32	2	452
FC08	2	452
LY-CoV016 (etesevimab)	3	417, 484
REGN10933 (casirivimab)	3	417, 484
REGN10987 (imdevimab)	6	446
C110	6	446
2H04	6	446

Table S2: Antibodies from other studies with neutralization profiles and sites of RBD escape that are similar to clusters of antibodies from out study. R1-32 and FC08 show profiles similar to cluster 2 [1, 2]; LY-CoV016 and REGN10933 show profiles similar to cluster 3 [3, 4]; REGN10987, C110, and 2H04 show profiles similar to cluster 6 [5].

tab:antibody˙escape

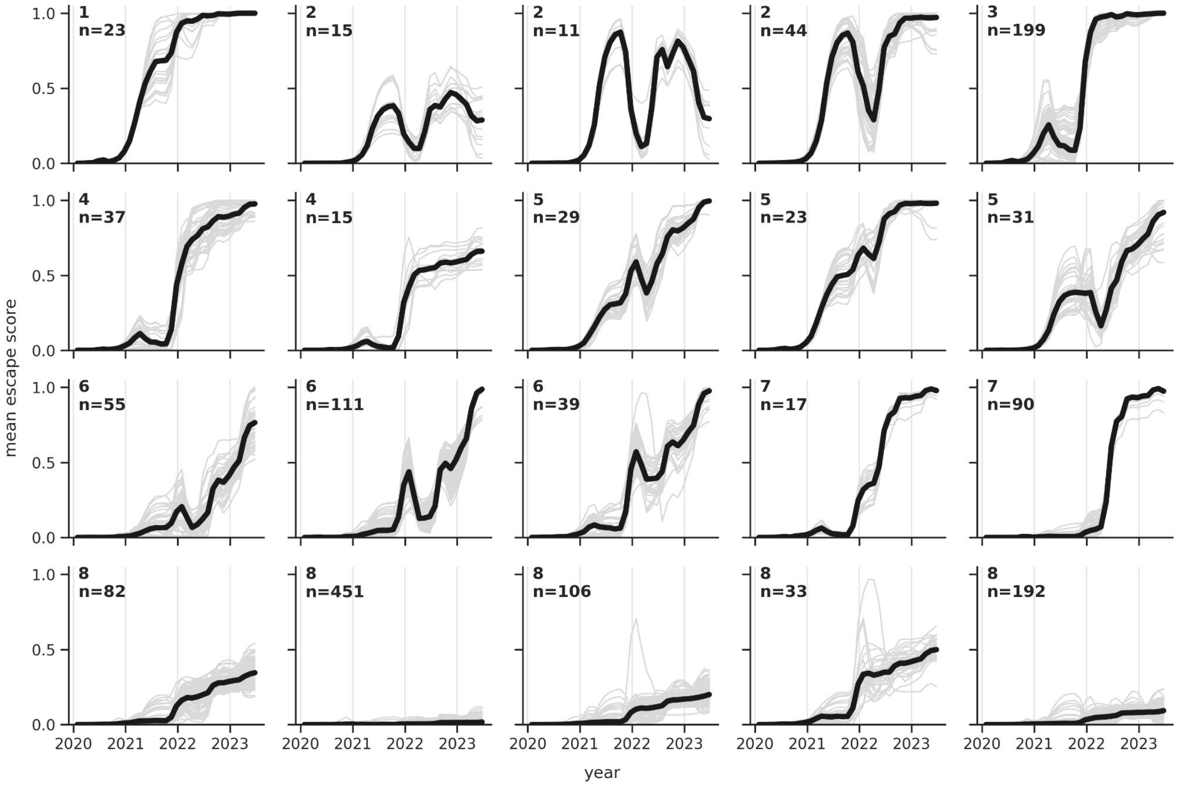

Figure S1: The 20 clusters of escape trajectories generated by k-means clustering. Each panel shows data for a different cluster. In each panel, thin gray lines show trajectories for individual antibodies within a cluster, while the bold black line shows the average trajectory. The title of each panel indicates the consolidated cluster number 1-8 from Figure 2B (e.g., the three panels with the title of "2" were all combined into a single cluster) as well as the number of antibodies per panel. fig:kmeans

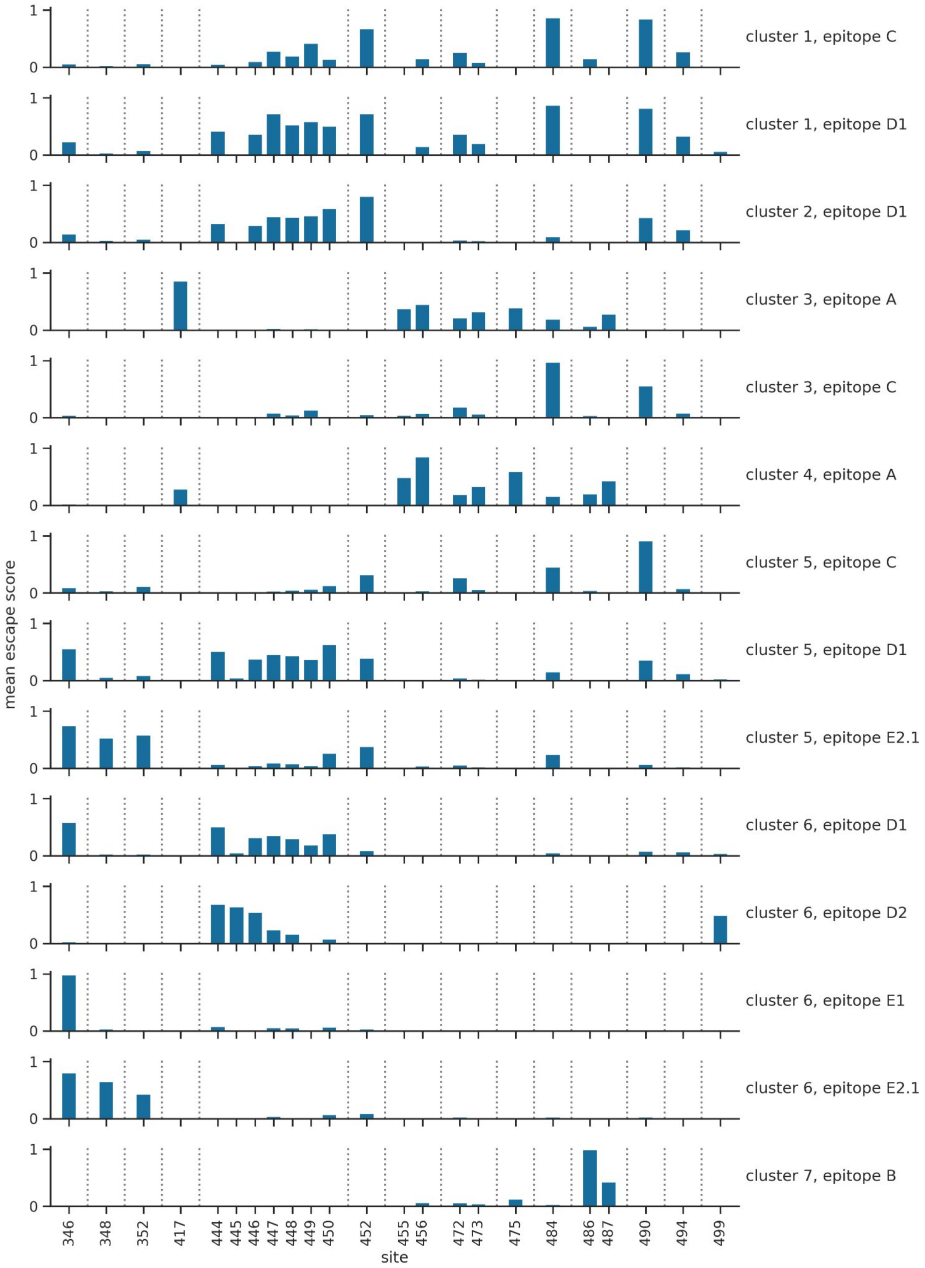

Figure S2: Average site-level escape scores of all antibodies in a given escape cluster and epitope. Escape clusters come from this paper, while epitope clusters come from Cao et al. [6]. The plot only shows data for sites where the average site-level escape score is >0.3 for at least one group of antibodies. fig:dms˙profiles

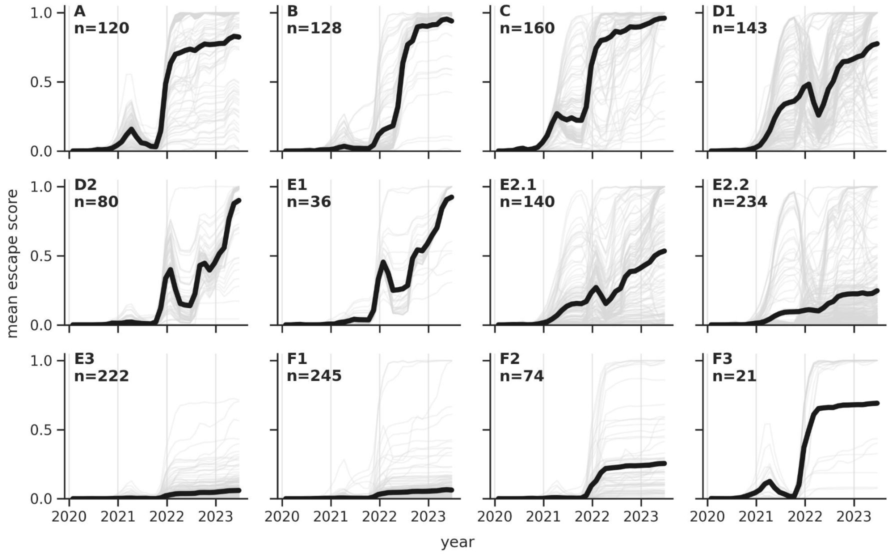

Figure S3: Escape trajectories clustered by antibody epitopes. Each panel shows data for a different epitope cluster, as defined by Cao et al. [6]. In each panel, thin gray lines show trajectories for individual antibodies within an epitope cluster, while the bold black line shows the average trajectory. The title of each panel indicates the epitope as well as the number of antibodies per panel. fig:trajectories˙by˙epitope

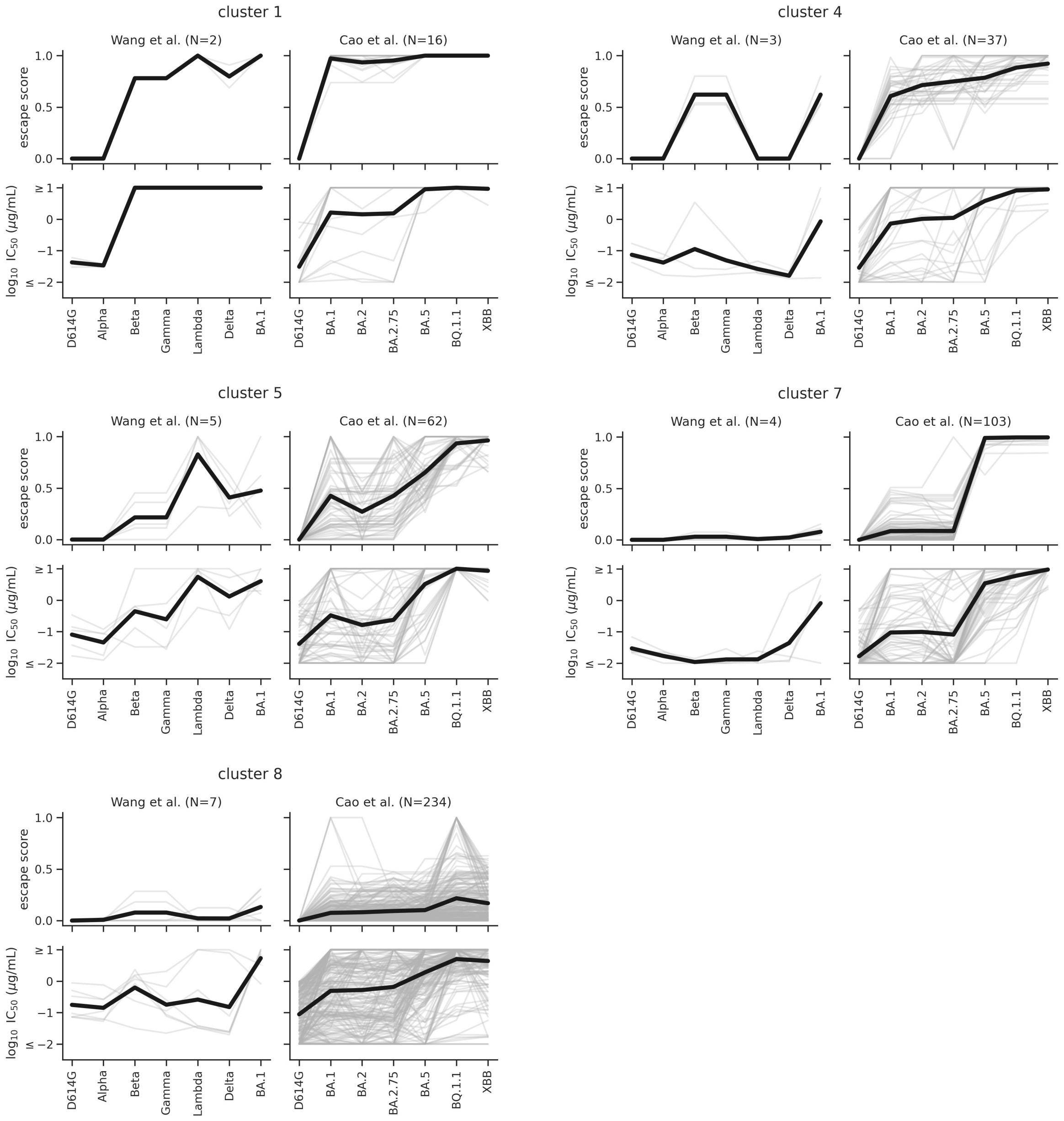

Figure S4: Comparing predicted escape scores and pseudovirus neutralization data for the remaining clusters. This figure is similar to Figure 4B-E, but shows data for the remaining clusters.

fig:validation˙supp

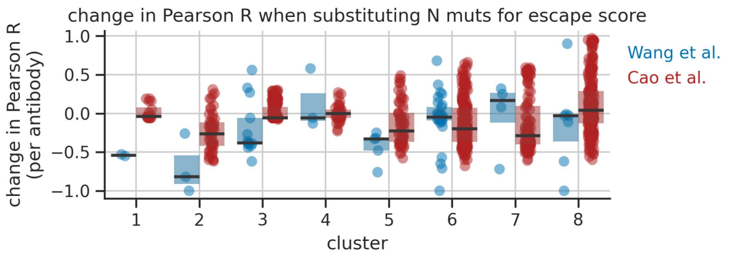

Figure S5: Escape scores capture more than sequence divergence. This figure is similar to Figure 4B, but shows the change in per-antibody correlation coefficients when they are computed using the number of RBD mutations in a spike variant rather than its predicted escape score for that antibody. Negative changes mean that the correlation coefficients are lower when using the number of RBD mutations. We impose a floor of -1 on these values. For clusters 1-3 and 5-7, the median correlation coefficient per cluster is substantially less than zero for at least one study. Note: in cases where the median change is close to zero, that does not necessarily indicate that the escape scores for that group of antibodies are incorrect, it merely indicates the pattern aligns with sequence divergence. fig:validation˙delta˙corr

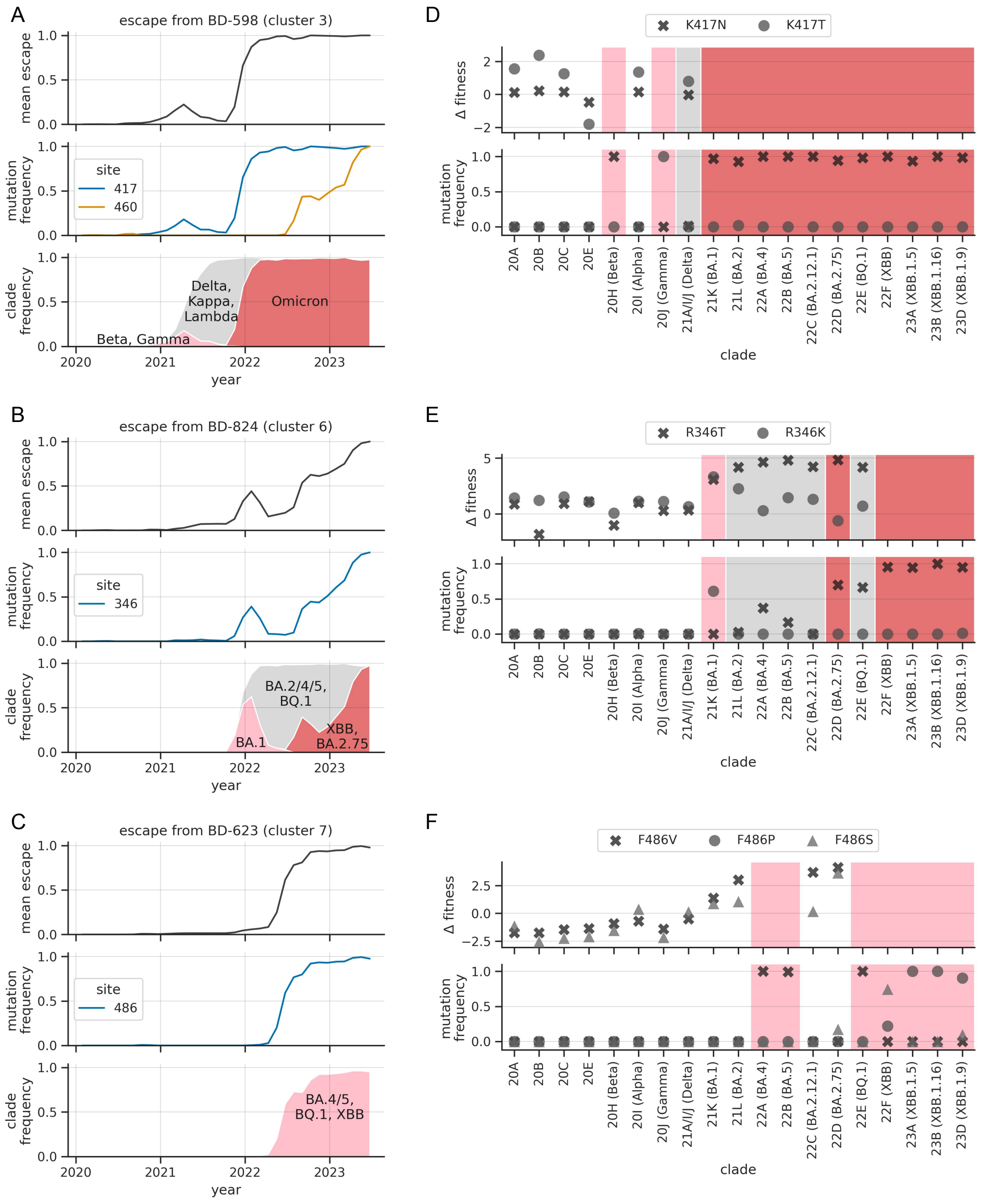

Figure S6: Same as Figure 5, but panels A-C show data for a different set of example antibodies and panels D-F show data for escape mutations associated with those antibodies.

fig:clade˙displacement˙supp

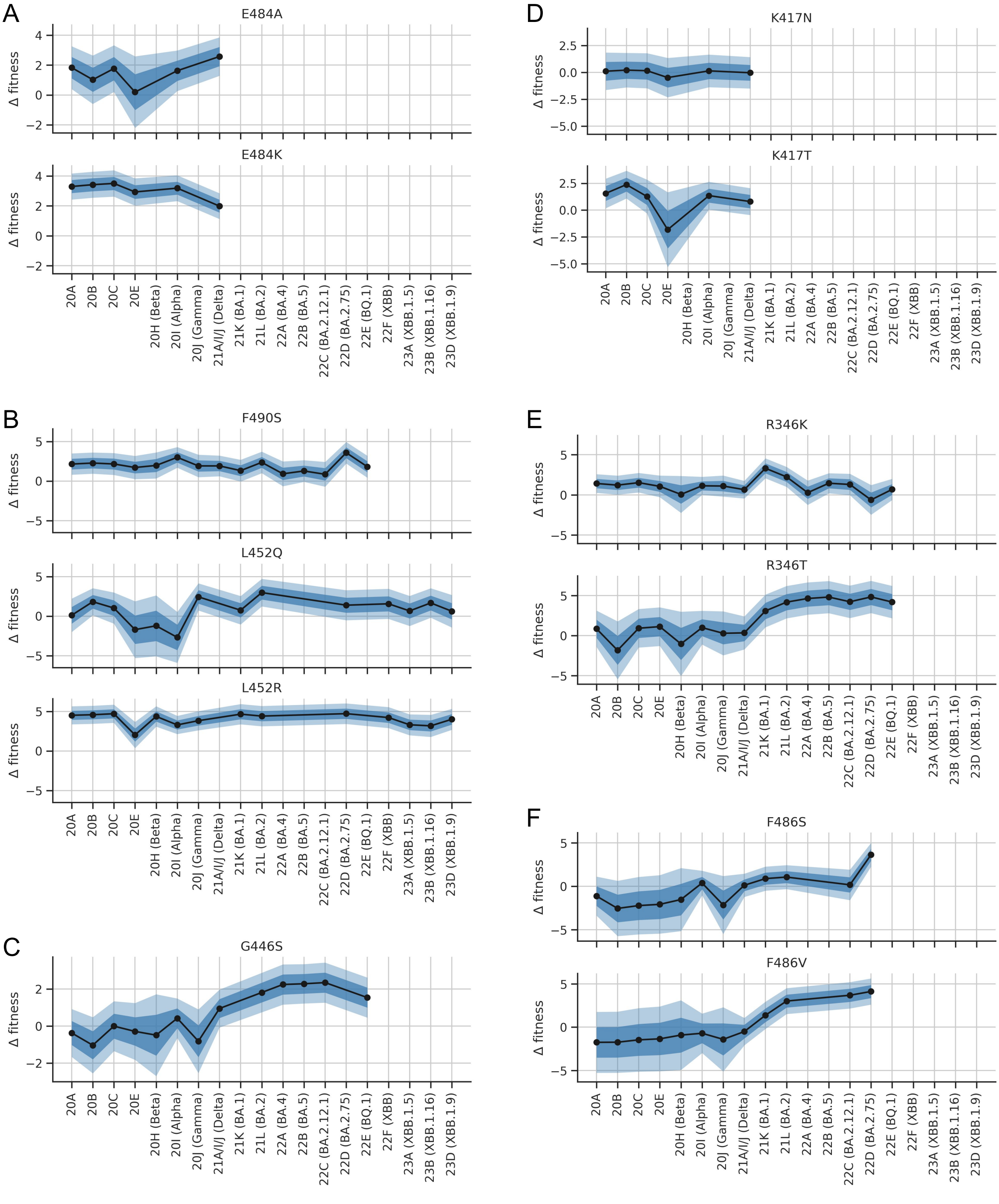

Figure S7: Levels of uncertainty in estimated fitness effects. Each panel shows fitness effects for a given group of mutations. Panels A-C show groups of mutations from Figure 5D-F, while panels D-F show groups of mutations from Fig. S6D-F. In each panel, each subplot shows data for a given mutation. For a given clade on the x-axis, the black dots show a mutation's fitness effect, computed as the mean of the posterior distribution over fitness effects, while the dark blue region extending above and below each dot shows the mean plus or minus the standard deviation of the posterior distribution. The light-blue region shows the mean plus or minus two times the standard deviation.

fig:fitness˙uncertainty

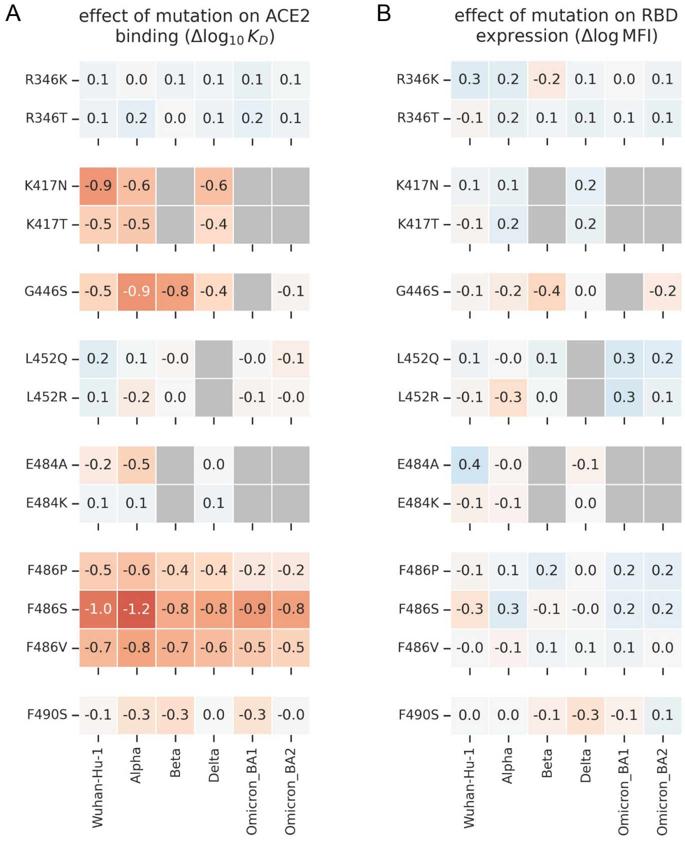

Figure S8: Effects of mutations on RBD-ACE2 binding and RBD expression. (A) Effects on binding. **(B)** Effects on expression. In both panels, the color scale ranges from −2 to 2, with negative numbers indicating that the mutation decreases binding or expression. These data come from two studies by Starr et al. [7, 8]. fig:rbd dms

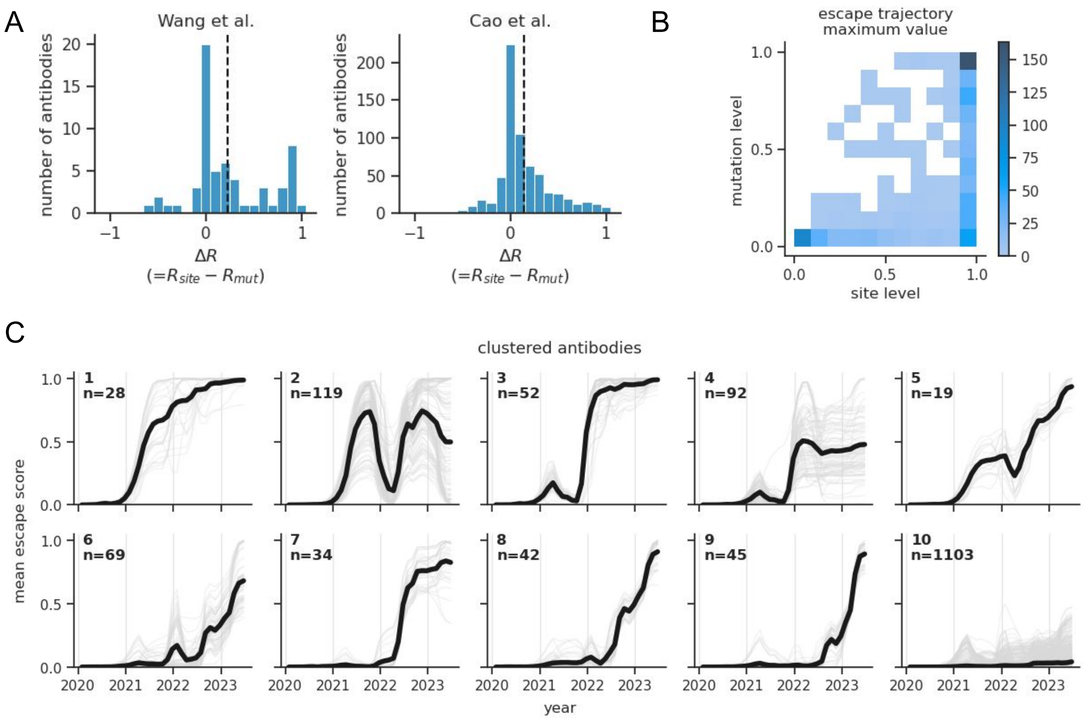

Figure S9: Comparing site-level and mutation-level approaches for computing viral escape scores. We tested two approaches to compute viral escape scores. The first approach computes scores using site-level $x_{r,a}$ values, as described in the Results. The second approach computes scores using mutation-level $e_{a,r,m}$ values, as described in the Methods. In principle, the mutation-level approach should be more accurate, since different mutations at the same site can have different effects. In practice, the site-level approach was better at predicting experimentally measured pseudovirus neutralization data, probably because the mutation-level $e_{a,r,m}$ values are noisy and site averaging corrects for that noise to some degree. **(A)** For each approach, we separately computed per-antibody Pearson correlation coefficients between predicted escape scores and measured $\log_{10} \mathrm{IC}_{50}$ values, as in Figure 4B. For each antibody, we then computed the difference between the correlation coefficients from each approach ($= R_{\mathrm{site}} - R_{\mathrm{mut}}$, where R_{site} and R_{mut} are coefficients for the site-level and mutation-level approaches, respectively), flooring coefficients at zero (negative values are adjusted to zero) before computing the difference. The left plot shows the distribution of differences for data from Wang et al., while the right plot shows this for data from Cao et al. Vertical dashed lines show the average of each distribution. In both studies, the site-level approach gave higher correlation coefficients, indicating this approach is more accurate at predicting the neutralization data. **(B)** We separately computed escape trajectories using each approach. This plot shows a 2D histogram comparing the maximum value of each trajectory when computed using the site-level approach (x axis) vs. the mutation-level approach (y axis), with the color bar indicating the number of trajectories in each bin. The maximum values are often lower when using the mutation-level approach, indicating less predicted escape. This difference appears to be one reason why escape scores computed using this approach do not correlate as well with the neutralization data. There are many antibodies for which the mutation-based viral escape score is low, but the experimentally measured escape is high (data not shown). **(C)** This panel is similar to Figure 2B, but shows trajectories computed using the mutation-level approach. We used k-means clustering to generate 20 clusters, then manually consolidated them into 10 clusters. The clustered trajectories showed qualitatively similar patterns to the ones in Figure 2B, though the precise number of antibodies per pattern was often different. This is partially because substantially more trajectories had low escape across all time windows (e.g., cluster 10 in this panel has >200 more antibodies than cluster 8 in Figure 2B). fig:site˙mut˙comparison

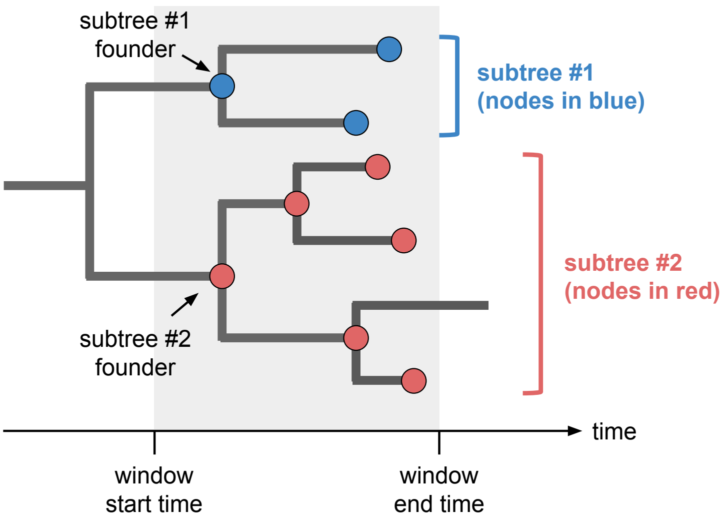

Figure S10: Strategy to identify subtrees in a sliding window. This figure shows a hypothetical time tree. The x axis gives the time of each node. The gray box shows a sliding window with a given start time and end time. There are two subtrees in the window. Arrows point to the founder node of each subtree. Blue circles show all nodes in the first subtree, and red circles show all nodes in the second. fig:sliding˙window

References

[1] Qingwen He, Lili Wu, Zepeng Xu, Xiaoyun Wang, Yufeng Xie, Yan Chai, Anqi Zheng, Jianjie Zhou, Shitong Qiao, Min Huang, et al. An updated atlas of antibody evasion by SARS-CoV-2 Omicron sub-variants including BQ.1.1 and XBB. *Cell Reports Medicine*, 4(4), 2023.

[2] Ping He, Banghui Liu, Xijie Gao, Qihong Yan, Rongjuan Pei, Jing Sun, Qiuluan Chen, Ruitian Hou, Zimu Li, Yanjun Zhang, et al. SARS-CoV-2 Delta and Omicron variants evade population antibody response by mutations in a single spike epitope. *Nature Microbiology*, 7(10):1635–1649, 2022.

[3] MacGregor Cox, Thomas P Peacock, William T Harvey, Joseph Hughes, Derek W Wright, Brian J Willett, Emma Thomson, Ravindra K Gupta, Sharon J Peacock, David L Robertson, et al. SARS-CoV-2 variant evasion of monoclonal antibodies based on in vitro studies. *Nature Reviews Microbiology*, 21(2):112–124, 2023.

[4] Meng Yuan, Xueyong Zhu, Wan-ting He, Panpan Zhou, Chengzi I Kaku, Tazio Capozzola, Connie Y Zhu, Xinye Yu, Hejun Liu, Wenli Yu, et al. A broad and potent neutralization epitope in SARS-related coronaviruses. *Proceedings of the National Academy of Sciences*, 119(29):e2205784119, 2022.

[5] Min Huang, Lili Wu, Anqi Zheng, Yufeng Xie, Qingwen He, Xiaoyu Rong, Pu Han, Pei Du, Pengcheng Han, Zengyuan Zhang, et al. Atlas of currently available human neutralizing antibodies against SARS-CoV-2 and escape by Omicron sub-variants BA.1/BA.1.1/BA.2/BA.3. *Immunity*, 55(8):1501–1514, 2022.

[6] Yunlong Cao, Fanchong Jian, Jing Wang, Yuanling Yu, Weiliang Song, Ayijiang Yisimayi, Jing Wang, Ran An, Xiaosu Chen, Na Zhang, et al. Imprinted SARS-CoV-2 humoral immunity induces convergent Omicron RBD evolution. *Nature*, 614(7948):521–529, 2023.

[7] Tyler N Starr, Allison J Greaney, William W Hannon, Andrea N Loes, Kevin Hauser, Josh R Dillen, Elena Ferri, Ariana Ghez Farrell, Bernadeta Dadonaite, Matthew McCallum, et al. Shifting mutational constraints in the SARS-CoV-2 receptor-binding domain during viral evolution. *Science*, 377(6604):420–424, 2022.

[8] Tyler N Starr, Allison J Greaney, Cameron M Stewart, Alexandra C Walls, William W Hannon, David Veesler, and Jesse D Bloom. Deep mutational scans for ACE2 binding, RBD expression, and antibody escape in the SARS-CoV-2 Omicron BA.1 and BA.2 receptor-binding domains. *PLoS Pathogens*, 18(11):e1010951, 2022.
